# Influence of Mothers/Grandmothers Coviewing Cartoons With Children on Children’s Viewing Experience

**DOI:** 10.3389/fpsyg.2020.01232

**Published:** 2020-06-18

**Authors:** Qi Meng, Xiaoying Sheng, Jiayin Zhao, Yifang Wang, Zhuqing Su

**Affiliations:** ^1^ Department of Teacher Education, Capital Normal University, Beijing, China; ^2^ Mental Health Prevention Hospital of Haidian District, Beijing, China; ^3^ Department of Psychology, Capital Normal University, Beijing, China; ^4^ Department of Preschool Education, Capital Normal University, Beijing, China; ^5^ Department of Preschool Education, Yichun Early Childhood Teachers College, Yichun, China

**Keywords:** cartoon, children, viewing experience, coviewing, mothers and grandmothers

## Abstract

Watching cartoons is one important event in children’s early lives. This activity is highly influential on many factors, such as children’s cognitive and behavioral development. Some researchers believe that parents should coview cartoons with children to help them filter and distinguish useful content. However, intergenerational education is already common in China, and the influence of grandparents cannot be ignored. Because they are in different stages of life, the members of these two generations manifest great differences in parenting style, which may lead to differences in child development. Does this generational difference have differential effects on the children’s cartoon-viewing experience? We recruited 89 parents and grandparents and their kindergarten-aged children (approximately 5 years old) to participate in the study. The mothers or grandmothers were asked to coview a cartoon for approximately 7 min with their child, after which the child was asked questions about the cartoon-viewing experience. The results show the following: (1) compared with grandmothers, mothers generally think that cartoons have a very high influence on children’s physical and mental health (*χ* ^2^ = 8.83, *p* < 0.05), (2) mothers place more restrictions on the content of cartoons that their children view, whereas grandmothers’ attitudes are characterized by greater tolerance (*χ* ^2^ = 11.94, *p* < 0.01), and (3) in the case of coviewing with mothers, when the children are asked “why” questions about the cartoon-viewing experience questionnaire, they use more experience proofs to explain their answers than when they coview with grandmothers (*χ* ^2^ = 16.69, *p* < 0.01; *χ* ^2^ = 10.44, *p* < 0.05).

## Introduction

Cartoons are very popular among children today because of their unique style and expression. Moreover, they exert great influence on children’s cognitive and behavioral development, as well as many other factors (Klein and Shiffman, 2006; Jiang, 2013; Habib and Soliman, 2015). In China, cartoons are diverse, and many are of high quality. High-quality cartoons convey positive information and useful knowledge, which is very helpful for children’s speech, logical thinking, positive emotions, and the formation of good values. Cartoons may even promote progress in childhood disease treatment (Schmidt and Vandewater, 2008; Stevenson, 2012; Frémont et al., 2018). Habib and Soliman (2015) have indicated that some prosocial plots in cartoons will help children learn social skills and practical life skills. For example, in classic cartoons from three different countries, *Dora the Explorer, The Smurfs*, and *Peppa Pig* (Carter, 2008; Chen, 2018), Dora’s active adventure seeking, the Smurfs’ bravery and wisdom, and Peppa’s family’s pleasant relationship are very effective demonstrations and hold positive educational significance for children, which in turn has a positive social impact. However, the overall quality of cartoons is mixed, and a few cartoons are laced with violence, foul language, and misguided behaviors. For example, many such qualities are evident in the plots of the Japanese cartoon *Ultraman*, the American cartoon *Happy Tree Friends*, and the Chinese cartoon *Boonie Bears*. *People’s Daily Online* reported that during the Spring Festival of 2016, a 10-year-old girl imitated the violent behavior of Logger Vick in *Boonie Bears* by sawing her sister’s nose with a chainsaw (“The chainsaw girl is impressed by the *Boonie Bears*, a cartoon grading system is imminent,” *People’s Daily Online*, March 10, 2016). According to George Gerbner’s definition, the harm behind this kind of media violence is not completely limited to behavior; it also causes psychological and emotional damage to people—especially to children, who are not fully developed.

Regardless of the type of cartoon watched, it will inevitably lead to an intuitive viewing experience for children. The viewing experience is a dynamic artistic experience involving emotional reactions and thoughts generated by children watching and enjoying art forms that express culture in visual formats, such as cartoons, television (TV) dramas, and movies, combined with their previous knowledge reserves (Eckhoff and Guberman, 2006). The cartoon-viewing experience includes several dimensions of individual cognitive development: identity, emotion, aggressive, fantasy, and reality (Bjorkqvist and Lagerspetz, 1985). Studies have shown that long-term watching of cartoons with negative content (violent, uncivilized behavior, etc.,) can lead to negative viewing experiences for children, such as generating more negative behaviors and emotions, increasing aggressive illusions, and thinking in ways that more easily diverge from reality. Finally, watching such cartoons affects development throughout one’s entire childhood (Bandura, et al., 1963; Hapkiewicz and Roden, 1971; Osborn and Endsley, 1971; Gunter and Harrison, 1991; Huesmann et al., 2003; Huang, 2016). A study by Prescott et al. (2018) supports this claim through a meta-analysis of 24 studies. The investigators note that playing violent video games can exacerbate children’s physical attacks over time. A longitudinal study by Huesmann et al. (2003) from 1977 to 1992 shows that excessive exposure to media violence during childhood can even predict early adult aggression. When children grow up, their increased negative behaviors will inevitably have a certain degree of bad influence on society. A meta-analytic study by Greitemeyer and Mugge (2014) also supports this conclusion.

In these viewing experiences, the study of children’s sense of reality is particularly important. Cartoons contain many illusory events, but young children cannot completely distinguish the illusory events in cartoons from real-life events (Nurşen, 2004). Children may learn illusory behaviors, make mistakes, and even engage in life-threatening behaviors; news reports on such behaviors are not uncommon. For example, *People’s Daily* reported that an 8-year-old girl in Chengdu learned rock climbing because she was imitating Briar and Bramble in the *Boonie Bears* (“*Boonie Bears* blamed for 8-year-old girl’s death? What should be blamed?”, China Youth Daily, August 01, 2018). Because of such potential for danger, distinguishing illusory from real events is an important cognitive task for preschoolers and a basic skill for daily activities. According to Woolley (1997), “reality” refers to things or phenomena that exist in our daily lives or are consistent with our real-life laws, such as airplanes, birthdays, going to the supermarket to buy things, etc., In contrast, “fantasy” refers to things or phenomena that do not exist in daily life or are inconsistent with our real-life laws—for example, a plot in *Tom and Jerry* in which, when Jerry was chased by Tom, his body was blown into a ball by the wind. Piaget posited that until the age of 12, children do not have the ability to distinguish between reality and unreality. A study by Harris and Koenig (2006) found that children aged 4–5 could not accurately judge illusory events and objects. Other researchers also believe that younger children often mistakenly judge real events as illusory; the ability to distinguish the two will gradually increase as children move into primary school (Martarelli and Mast, 2013; Li et al., 2015; Martarelli et al., 2015; Maftei and Măirean, 2017). Moreover, this kind of ability is also related to children’s judgment and logical thinking. Some previous studies have found that children’s ability to distinguish illusory events from real-life events is likely to be shaken by what they have seen (Chandler and Lalonde, 1994; Rosengren and Hickling, 1994; Subbotsky, 2004). In such studies, even though the children believed at first that an event was an illusory event and would not happen in real life, more than half of the children changed their original judgment and logical thinking when they saw the magical illusory event happening in front of their eyes. Some children used factual information such as experience proofs to explain the reason for their change in judgment, for example, “This is not a scam, because I saw it with my own eyes.” Other children explained with simple information, such as redundant proofs, for example, “It just happened.” Others rationalized their explanations with “if” clauses such as hypothesis proofs, for example, “If I don’t see this, I may not believe it” (Chandler and Lalonde, 1994; Shtulman and Carey, 2007). This easy change in judgment also illustrates the instability of preschool children’s distinctions between illusory events and real-life events as well as the development of judgmental thinking. Piaget noted that this confusion can hinder children’s cognitive development; thus, consumption of too much illusory content is not recommended for children.

Based on this consideration, the American Academy of Pediatrics Committee on Public Education (2001) suggested that parents should accompany their children when watching cartoons, guiding their children and explaining the cartoon content. This approach can effectively reduce the negative impact of cartoons’ bad content on children. Although individual researchers have proposed opposing views (Dorr et al., 1989; Skouteris and Kelly, 2006; Collier, et al., 2016), many previous studies have shown that parental coviewing can not only reduce the emergence of children’s negative emotions caused by panicky, suspenseful, and other TV footage (Wilson and Weiss, 1993; Nathanson and Cantor, 2000; Linder and Werner, 2012) but also promote preschoolers’ learning (Rice et al., 1990; Sims and Colunga, 2013; Strouse et al., 2018) and improve their understanding of TV content (Morgenlander, 2010). According to İvrendi and Özdemir (2010), when a mother and a child watch TV together, the child’s thinking is more easily diverted from reality, such that the child confuses the real and illusory worlds in the cartoons. Therefore, for children’s development, reducing the time that children spend watching TV programs and supervised viewing are usually advocated.

To date, intergenerational education is very common in China. Therefore, the influence of grandparents on children should not be ignored. Many studies have found that mothers and grandmothers’ parenting status is an important indicator of children’s well-being and physical and mental health (Dolev and Habib, 1997; Socolar, 1997). However, mothers and grandmothers exhibit great differences in their parenting styles and how they interact with children (Staples and Smith, 1954; Stevens, 1984; Smith, 1991; Chase-Lansdale et al., 1994; Ruiz and Silverstein, 2007). For instance, Xing et al. (2012) found several intergenerational differences between Chinese mothers’ and grandmothers’ parenting behaviors: for example, mothers provide more advice, guidance, explanation and reasoning, positivity, and evaluation to achieve specific parenting goals in the process of raising children than do grandmothers. Mothers also place more emphasis on the development of children’s independent socialization goals than do grandmothers. In the process of getting along with children, mothers show more emotional support and school involvement, while grandmothers are mainly concerned with the child’s life and physical health (Coyl-Shepherd and Newland, 2013; Xing et al., 2016). This difference may lead to variations in the level of their participation in children’s daily lives and have a major impact on the children’s social development (Xing et al., 2016). In addition to myriad demographic variables, children’s personality factors, etc., this difference may also be caused by children’s different behavior patterns learned in the contexts of the two generations. Compared with grandmothers, mothers may pay more attention to children’s education and quality of life, and thus, they are willing to spend more time with children to learn or participate in parent-child interactions. According to the observational learning theory, through such role modeling, positive behavior can be quickly and directly learned by children to minimize the negative impact of harmful external information. In contrast, parents’ bad behavior and negative parent-child relationship patterns will also quickly be absorbed by the child, which is not conducive to children’s social development.

In summary, previous studies, domestic and abroad, have analyzed and explained cartoons from different perspectives. However, some studies have simply discussed the impact of cartoons or media use on children without considering the roles and influences of parents (Bandura et al., 1963; Hapkiewicz and Roden, 1971; Osborn and Endsley, 1971; Gunter and Harrison, 1991; Huesmann et al., 2003; Carter, 2008; Schmidt and Vandewater, 2008; Stevenson, 2012; Huang, 2016; Frémont et al., 2018). Some studies explore the impact of parents on child growth but do not compare the influence of grandparents (Dorr et al., 1989; Rice et al., 1990; Wilson and Weiss, 1993; Nathanson and Cantor, 2000; Skouteris and Kelly, 2006; İvrendi and Özdemir, 2010; Morgenlander, 2010; Linder and Werner, 2012; Sims and Colunga, 2013; Collier et al., 2016; Strouse et al., 2018). Some studies compare the effects of intergenerational differences on children’s education and growth but have not considered whether such differences also have differential effects on children’s daily entertainment activities (Staples and Smith, 1954; Stevens, 1984; Smith, 1991; Chase-Lansdale, et al., 1994; Ruiz and Silverstein, 2007; Xing et al., 2012, 2016; Coyl-Shepherd and Newland, 2013). Therefore, the aims of this study are to explore the attitudes of mothers and grandmothers toward children watching cartoons and further explore the impact of intergenerational differences on children’s viewing experiences. The study of intergenerational differences is necessary in China, because, given the rapid development of Chinese society; there are an increasing number of families in which elder members usually take care of the children. Generally, women (mothers and grandmothers) put more energy into raising children. The two generations inevitably have different opinions in this regard. Thus, improving ways of educating and raising children have become a topic of immense concern to parents and have also become a primary issue that is closely connected with social development. After all, children’s healthy growth is the foundation of social development, and family education is undoubtedly an indispensable element of this foundation.

The results of this study are derived from the professional perspective of psychology. Parents can obtain reference value from the results, change their parenting style and companionship modes, and re-examine their parent-child relationship. In addition, parents can learn to better screen cartoons that are helpful to children and learn how to view the cartoons with them. It is better to accompany children when they are watching cartoons. In addition, the results of the study may also affect the teaching mode of some kindergarten teachers, who may communicate their knowledge by watching cartoons with the children and explaining the patterns that occur. Parents and teachers who feel that this change is effective will personally recommend it to other parents and teachers. This spontaneous transmission may have a certain social impact in the education sector. In addition, this study may also play a role in the establishment of a normative grading system of domestic animation content and enrich the research in related fields in China.

Hence, we propose the following hypotheses:

1. Mothers and grandmothers may have significant differences in their attitudes toward viewing cartoons.
2. Mothers and grandmothers have significantly different effects on children’s cartoon-viewing experience.

## Materials and Methods

### Participants

In this study, 89 children (including 49 boys) and their parents/grandparents (including 49 mothers) were selected from three different classes of two public kindergartens in Beijing. The average age of children was 70.55 ± 4.94 years. The average age of the mothers was 36.53 ± 3.25 years; the average age of the grandmothers was 63.58 ± 5.16 years. Among participants, 46 mothers and 16 grandmothers were educated at the university level; 33 mothers had annual incomes of more than 100,000 yuan, as did two grandmothers. We recruited the mothers (grandmothers) of children in a senior kindergarten class because children in this age group are at the preschool stage, which is an important learning stage prior to elementary school. We were particularly eager to learn whether the overall attitudes of mothers and grandmothers affected their children.

### Experimental Design

This study used a single-factor (mothers vs. grandmothers) between-subjects design to explore whether there would be a difference between the influence of mothers and grandmothers coviewing cartoons with their children/grandchildren on the children’s cartoon-viewing experience. We adopted the design concept of a natural experiment method. After strict control of the variables, we used this more socially oriented experimental design to improve the ecological validity of the experiment and to ensure that it would be consistent with the real living environment. This approach is more authentic, universal, and applicable than laboratory experiments.

### Procedure

Adult participants were recruited through telephone calls and flyers posted at the kindergarten. Child participants were also recruited at the same kindergarten and were selected based on adult participants. Before the experiment began, we asked mothers/grandmothers to provide written informed consent to ensure that they knew what they were doing and what their children/grandchildren were going to do. We also informed them of the voluntary and confidential nature of the study. The process of data collection was approved by the Ethics Committee of the Psychology Department of Capital Normal University.

First, the mothers/grandmothers were asked to enter an empty classroom with their children/grandchildren. Next, they were instructed to watch a cartoon clip for approximately 7 min in a relaxed state with only the children present. After the mothers/grandmothers explicitly requested to adjust their seated position and the computer, the experimenter clicked the “start video” button and left the classroom, leaving only one mother/grandmother and child. After approximately 10 min (or less than10 min if the participants took the initiative to tell the experimenters that they had finished coviewing), the experimenter returned. Then, the experimenter asked the mothers/grandmothers to leave and sat next to the child. To ensure objectivity, the experimenter used a computer to play the recording of the questions in turn and asked children to answer. The whole process was recorded using a mobile phone.

### Experimental Materials

#### Demographic Questionnaire

With the help of each kindergarten teacher, the experimenter who was involved issued a demographic questionnaire to all adult participants, and the objectives as well as the questionnaires were briefly explained to all parents. All of the mothers had normal vision; grandmothers with vision problems were helped by an investigator when answering the questionnaire. The questionnaire included children’s names, ages, and genders; parents’ ages, levels of education, and annual income were also included. We added the following three questions to the end of the questionnaire: “Do you think that your child’s cartoon viewing significantly influences him or her?”, “Do you limit your child’s cartoon-viewing time?”, and “Do you monitor the content of the cartoons that your child views?” (see the full questionnaire in Appendix 1).

#### Boonie Bears Video Clip

Boonie Bears is a Chinese cartoon created specifically for children. This cartoon tells the story of two bear brothers (Briar and Bramble) who work together to deal with a bald lumberjack (Logger Vick) and protect the virgin forest. The reason for choosing this cartoon was that it was broadcast on CCTV and various online media after its release in 2013, and its ratings exceeded those of the famous Chinese cartoon Pleasant Goat and Big Wolf. The clip was selected from the fourth episode of *The Big Adventures of the Bears*. The total length of the episode was 13 min. The end credits and parts of the episode not relevant to the plot were removed, with 7 min and 30 s remaining.

#### Cartoon Playback Device

A computer with a 24-inch screen was used to play the cartoon video clip.

#### Children’s Cartoon-Viewing Experience Questionnaire

With reference to the research of Bjorkqvist and Lagerspetz (1985) on measuring children’s cartoon-viewing experience, we compiled a questionnaire. The questionnaire included a total of 16 questions representing the following five dimensions: understanding, identity, emotion, reality, and aggression. Regarding the coding of the “why” questions in the “reality” dimension, we refer to the coding system of Shtulman and Carey (2007) and divide the children’s reasoning types into four categories: (A) *Experience proofs*, (B) *Hypothesis proofs*, (C) *Redundant proofs*, and (D) *Do not know*. *Experience proofs* referenced facts about the world that would preclude an event’s occurrence (e.g., “There are no wooden ladders in the world, only iron ladders”; “Normal people are not so heavy, they wouldn’t break the ladder”). *Hypothesis proofs* referenced hypothetical events that could occur, or would occur, in place of the actual event under consideration (e.g., “If you want to eat honey, you can go to the supermarket to buy it”; “People don’t run on rockets unless they have poor eyesight”). *Redundant proofs* provided no information beyond what was already mentioned in the story or what was already discernible from a participant’s initial judgment (e.g., “that’s not possible,” “that’s not real,” “it can only happen in stories”). Some examples of the questions in the questionnaire and coding scheme are shown in Table 1 (see the full questionnaire in Appendix 2).

**Table 1:** Examples the children’s cartoon-viewing experience questionnaire.

Dimension	Question	Coding scheme
Reality	8. To eat honey, Briar and Bramble went to climb the tree and set the fire. Do you think this could happen in real life? Why?	**Do you think this could happen in real life?** **A**. Yes (1 point) **B**. No (2 points) **C**. “I do not know” or “I am not sure” (3 points) **Why?** **A**. **Experience proof** (1 point) **B**. **Hypothesis proof** (2 points) **C**. **Redundant proof** (3 points) **D**. **Do not know** (4 points)

### Data Processing

For the data from the demographic questionnaire, SPSS 22.0 version was used for statistical analysis processing. To record the questionnaire’s answers, we first compiled the records and then developed the coding table. We first selected the appropriate questionnaires and measurement dimensions according to the selected cartoons and the research of Bjorkqvist and Lagerspetz (1985). Then, we adjusted the questions according to the content of the cartoons and, finally, set the scoring criteria. After we determined the code list (see Appendix 2), 30 of the recordings were independently coded by two psychology graduates familiar with the coding rules. The scorer consistency coefficient (kappa coefficient) was 0.84.

## Results

### Analysis of the Differences Between Mothers’ and Grandmothers’ Attitudes Toward Children Viewing Cartoons

Taking the mothers/grandmothers as independent variables and their attitude toward children viewing cartoons (see Appendix 1) as a dependent variable, we used the chi-square test to analyze the data. The results showed significant differences between attitudes toward viewing cartoons (*χ* ^2^ = 8.83, *p* < 0.05) and between limitations on the content of cartoons (*χ* ^2^ = 11.94, *p* < 0.01). A belief that cartoons have a very high influence on children’s physical and mental health was endorsed by 85.7% of the mothers. In addition, 79.6% of the mothers reported frequently and strictly limiting the content of their children’s cartoon viewing. However, 87.1% of grandmothers thought that the cartoons had little effect on the children. Almost half of the grandmothers had stricter limitations on the content of cartoons that their grandchildren view; the other half held the opposite attitude (see Table 2).

**Table 2:** Chi-square test of mothers’/grandmothers’ attitudes toward the children’s cartoon-viewing experience (*χ* ^2^).

Parents’ identity	Questions	P5*^a^*				P6*^b^*				P7*^c^*			
	Answer type	a^1^	b^2^	c^3^	d^4^	e^5^	f^6^	g^7^	h^8^	e^5^	f^6^	g^7^	h^8^
Mothers		0	7	32	10	14	28	7	0	17	22	9	1
Grandmothers		2	13	21	3	16	22	1	1	9	11	11	9
*χ*^2^		8.83^*^				5.50				11.94^**^			

a Do you think viewing cartoons has an influence on children’s physical and mental health?

b Do you limit your child’s cartoon-viewing time?

c Do you monitor the content of the cartoons that your child views?

1 No influence.

2 Little influence.

3 More influence.

4 Very high influence.

5 Strict limit.

6 Often limit.

7 Low limit.

8 No limit.

* *df* = 3; *p* < 0.05;

** *df* = 3; *p* < 0.01.

### Analysis of the Differences in the Influence of Mothers and Grandmothers on Children’s Cartoon-Viewing Experience

A binomial distribution test was performed to determine “whether the children think the event in the cartoon could occur or not,” with the “reality” dimension serving as an independent variable (Q8-1, Q9-1, and Q10-1). According to the results, more than 80% of children believed that these three types of illusory events in a cartoon would not happen in real life. The probability of children’s responses was significantly different from the expected probability (*p* < 0.001), indicating that the child’s answer probability did not conform to the binomial distribution and was not a random guess (see Table 3).

**Table 3:** Probability test of whether children think that illusory events will occur (*p*).

Answer type	Questions		
	Q8-1^***^ *^a^*	Q9-1^***^ *^b^*	Q10-1^***^ *^c^*
Yes	0.84	0.82	0.94
No	0.16	0.18	0.06

a To eat honey, Briar and Bramble went to climb the tree and set the fire. Do you think this could happen in real life?

b When Bramble was chased by a bee, he ran away on a ladder that had been broken into two. Do you think this could happen in real life?

c Logger Vick used a rocket to shoot Warren into the sky. Do you think this kind of thing could happen in real life?

*** *p* < 0.01.

The mothers/grandmothers were taken as independent variables, the question-answer type of the four dimensions in the children’s cartoon-viewing questionnaire was the dependent variable, and the chi-square test was used to analyze the data. The results showed that mothers and grandmothers had significantly different influences on the children’s answer types for Q8-2 (*χ* ^2^ = 16.69, *p* < 0.01) and Q10-2 (*χ* ^2^ = 10.44, *p* < 0.05) in the reality dimension. In the case of mothers and children coviewing the cartoon, the number of children who answered Q8-2 using the *Experience Proof* answer type was highest, accounting for 75.6% of the children. The number of children who answered Q8-2 using the *Hypothesis Proof, Redundant Proof*, and *Do not Know* answer types was small. In the case of grandmothers and children coviewing the cartoon, the percentages of children who answered Q8-2 using the *Experience Proof*, *Hypothesis Proof*, and *Redundant Proof* answer types were approximately 34.4, 31.3, and 34.4%, respectively. In the case of mothers and children coviewing the cartoon, the percentage of children who answered Q10-2 using the *Experience Proof* answer type was highest, and the percentage of children using the *Redundant Proof* answer type was second highest, at 54.3 and 41.3%, respectively. The number of children who answered Q10-2 using the *Hypothesis Proof* and *Do not Know* answer types represented the minimums. In the case of grandmothers and children coviewing cartoons, the number of children who answered Q10-2 using the *Experience Proof* answer type was greatest, accounting for 54.3% of the variance, followed by children who used the *Hypothesis Proof* and *Redundant Proof* answer types (see Table 4). There was no significant difference between mothers and grandmothers with respect to the impact of children’s responses on other dimensions.

**Table 4:** Chi-square test of the different influences of mothers/grandmothers on children’s cartoon-viewing experience (*χ* ^2^).

Parents’ identity	Items	Q8-2*^a^*				Q9-2*^b^*				Q10-2*^c^*			
	Reason type	A^1^	B^2^	C^3^	D^4^	A^1^	B^2^	C^3^	D^4^	A^1^	B^2^	C^3^	D^4^
Mothers		31	3	5	2	30	11	5	2	19	1	25	1
Grandmothers		11	10	11	0	13	15	7	0	8	8	19	0
*χ*^2^		16.69^*^				7.83				10.44^*^			

a “Why” did you give your answer to Q8-1?

b “Why” did you give your answer to Q9-1?

c “Why” did you give your answer to Q10-1?

1 Experience proof.

2 Hypothesis proof.

3 Redundant proof.

4 Do not know.

* *df* = 3; *p* < 0.05.

## Discussion

### Differences Between Mothers’ and Grandmothers’ Attitudes Toward Children Viewing Cartoons

The significant differences in attitudes between mothers and grandmothers toward the children’s cartoon viewing are consistent with the results of previous studies. Barkin et al. (2006) argued that parents are more restrictive and more cautious about children’s cartoons than other caregivers because parents have a stronger sense of prevention against the negative effects of violence or other undesirable cartoon content. This discrepancy may be because the mothers and grandmothers had different styles of thought for their parenting methods. Because they held an important role in disciplining their children, the mothers were more concerned with whether viewing cartoons helped their children’s learning and physical and mental development. They believed that excessive viewing of cartoons would affect their children’s vision and physical exercise as well as the quality of their parent-child relationships (Silje et al., 2018; Webster et al., 2019). Therefore, most mothers had very strict restrictions on the content of TV programs (İvrendi and Özdemir, 2010). However, the grandmothers believed that they used to watch TV in the past and did not encounter any bad influences. Soumya et al. (2014) reported that some grandparents use TV as a “temporary babysitter,” and children watch TV when their grandparents are busy with other household chores. In contrast to parents, grandparents feel that children’s cartoon viewing can help them reduce the burden of caring for the children and bring the children joy. Second, studies by Thomas (1986) and Fuller-Thomson and Minkler (2001) have found that younger grandparents (40–60 years old) are more willing to participate in the daily life of grandchildren than older ones (70–80 years old). Furthermore, they are more willing to make useful recommendations for parenting. This difference may indicate that our results could be related to the age of the caregivers. The gradual aging of the grandparents’ physical functions, along with the various physiological and psychological illnesses that they may have, could mean they have too little energy to guide and participate in children’s daily activities (Kong and Wang, 2013). However, this possibility should be explored, specifically in our study.

### Differences in the Influences of the Mothers and Grandmothers on the Children’s Cartoon-Viewing Experiences

For more than 80% of the children, the illusory events portrayed in the cartoons were not expected to occur in real life, consistent with findings from previous studies. As Shtulman and Carey (2007) mentioned in their research, if children cannot imagine how an illusory event happened beyond their cognitive domain, then they will inevitably judge that illusory event could not occur in real life because there are two criteria by which children judge illusory and real events: whether events comply with the laws of physics and whether they comply with social rules (Nicholls and Thorkildsen, 1988). Events such as “some object floats in the air” in cartoons do not conform to the laws of physics. Therefore, this finding may be related to the criteria for child judgment. Second, many previous studies have found that children can accurately distinguish illusory events from real-life events at the age of 5–6 and that this ability generally improves with age and can even reach an adult level by the age of 7–8 (Harris et al., 1991; Johnson and Harris, 1994; Martarelli and Mast, 2013; Li et al., 2015; Martarelli et al., 2015; Maftei and Măirean, 2017). The child participants in this study were approximately 5 years old, and their cognitive development was transitioning from the previous stage of operation to the specific stage of operation. Their ability to observe and judge things is constantly changing, which is why they could more accurately judge that illusory events will not occur in real life. Third, Prentice et al. (1978) and Woolley et al. (2004) found that most preschoolers are more willing to believe that the beautiful and positive illusory characters, and illusory plots in cartoons will exist or occur than normal plots. The illusory events of cartoons in this study were relatively negative; thus, the children may have thought that they would not happen based on their subjective emotional experience. This result may be related to the type of illusory event in the cartoon.

When the children coviewed the cartoon with their mothers, they more commonly used the experience proof type to answer the “Why” question (Q8-2, Q9-2, and Q10-2) for the answers to Q8-1, Q9-1, and Q10-1. This result is consistent with that of previous research. Research has shown that adults have more mature logical thinking and reasoning skills due to their richer experience and knowledge. When children ask for reasons, parents generally use experience proofs to explain their reasoning more often than do children (Li et al., 2015). Under the influence of this thinking mode, parents can effectively impart their own past experience and knowledge when coviewing TV shows with their children, helping children distinguish illusory events from real-life events (Logan and Moody, 1979; Morgenlander, 2010). In this study, the greatest use of the experience proof type occurred for the children who coviewed with their mothers, indicating that the mother’s time spent on the child’s daily activities was being used efficiently. Mothers are good at teaching and learning, and they can find appropriate opportunities to educate children while viewing cartoons. This educational method includes how to distinguish between reality and illusory events in cartoons. Children are obsessed with their mother’s experience, their daily experiences, and accumulated knowledge; thus, mothers’ greater levels of experience can be used to explain the children’s questions. This finding shows that the children’s own experience and the knowledge provided by their mothers are both indispensable, which coincides with Vygotsky’s sociocultural cognitive theory (Vygotsky, 1978). He believed that the individual’s prior knowledge experience (e.g., that which their mothers imparted) acts as an intermediary, enabling individuals to establish new connections when viewing works of art (e.g., cartoons), thereby generating new understanding and thinking. The sociocultural and external educational environments will promote the cognitive development of individuals and eventually form a sound cognitive system. In addition, the average age of the children in this study was 5–6 years, and their theory of mind development was appropriate. The “Theory of Mind” (ToM) refers to an individual’s reasoning or cognition of the psychological state of oneself and others, as well as the individual’s relationship with others (Gopnik and Astington, 1988). Children’s ToM begins to form at 4 years of age (Freeman and Lacohee, 1995; Carlson et al., 1998). Once this ToM is formed, children can “pretend” that they are others and think differently, can see the motives and results of others’ behaviors, and distinguish between appearances and facts. Therefore, the children in this study could correctly distinguish the illusory and real events in the cartoon and use their daily learned experiences and the experiences taught by their mothers to give reasonable explanations.

When the children coviewed cartoons with their grandmothers, the numbers of children using experience proof, hypothesis proof, and redundant proof explanations were similar, consistent with the findings of previous studies. Some researchers believe that grandmothers are more willing to use simple and rude instructions, rewards, and cold treatments to raise children than mothers (Stevens, 1984; Smith, 1991) and do not pay much attention to communication and education with children. Moreover, most of the Chinese grandmothers’ disciplinary style in this study tended to be nanny-related, in which they were more concerned about the safety, food, and clothing problems of their grandchildren at home. This type of discipline will reduce the possibility of spreading useful knowledge and experience (Soumya et al., 2014; Xing et al., 2016). As a result, the grandmothers in this study did not pay as much attention to the content of cartoons when accompanying their grandchildren but directed more energy to the children’s needs and providing them with services. Even if the grandmothers participated in the interactive process of viewing cartoons, they reminded their grandchildren to pay attention *via* sight only. For other aspects of viewing, they did not care very much, nor did they offer any experiential education. The children in this study had developed an age-appropriate ToM, and they could essentially distinguish between real and illusory events. However, these children did not gain much experience from their grandmothers. Coupled with the lack of accumulation of children’s own daily experience, this experience may have led to a diversified thinking pattern. Furthermore, as mentioned above, there are two main methods for judging the likelihood of an event in addition to the method taught by adult experience. First, children may try to identify the conditions that allow the event to occur, and if they cannot, they will judge the event as impossible. We call this type the “hypothesis proof.” Second, children may not have tried to model each event but instead rely solely on their experience. We call this type the “redundant proof” (Li, 2014). Therefore, it is easy to understand that when the grandmother’s experiences were not adequately taught, the types of responses the child would offer as answers were diversified.

### Educational Advice

Based on the above findings, we found that the mothers were more rigorous about the children watching cartoons than were the grandmothers. Additionally, when watching cartoons with a mother, the child’s thinking in his or her answers became more divergent and also cited experience as evidence. These results should cause widespread concern in the education industry and cause parents to pay attention to their children’s education. Correspondingly, the division of responsibilities for caring for children in many Chinese families can also be appropriately changed. Due to the important influence of parents on children (Dolev and Habib, 1997; Socolar, 1997), we can appeal to parents to spend more time with their children at home and to participate in parent-child activities as much as possible to train their children’s cognitive development. Parents should be primarily responsible for educating their children and spreading knowledge (Mccarthy et al., 2003). They should not be so busy with work that they neglect to communicate with their children or push the task of educating their children to grandparents. An educational model in which parents are responsible for their children’s education will help children’s thinking development and experiential learning. In addition, grandparents should learn from this result. The interaction between grandparents and children should not always be like that of a babysitter (Hank and Buber, 2009). Grandparents should pay more attention to educating the children. In cases where their educational abilities do not allow such participation, they can take care of their grandchildren’s diets while their parents are busy (Hayslip et al., 2003). However, they should not live with their grandchildren for a long time because of their own needs. Instead, they should let the parents take primary responsibility for early education (Finch and Mason, 1991). In addition, teachers’ teaching methods can also be adjusted appropriately. Teachers cannot simply rely on the knowledge of books but must also spread knowledge through animation, video, etc., (Wang, 2008). Perhaps this approach will help children understand knowledge faster and better and help them generate their own new thinking and more angles from which to answer questions. If all the above suggestions can be implemented, cartoons will become a good assistant with which to help children learn and grow together with their parents.

## Limitations

This study revealed interesting results; however, certain limitations remain.

First, the sample size was small, which might lead to poor generalization of the results and negatively affect external validity.

Second, this design of this study was cross-sequential. In the future, a longitudinal study can be conducted to track the impact of children’s age development trends on the results of the study, starting with children in junior classes.

## Conclusion

Compared with grandmothers, mothers pay more attention to the influence of cartoons on their children’s physical and mental health, so the limitations that mothers place on cartoon content are stricter. In general, mothers have more influence on their children’s cartoon-viewing experience than do grandmothers. In the case of coviewing cartoons with their mothers, children are more likely to use their accumulated knowledge and experience to think and answer questions.

## Data Availability Statement

The datasets generated for this study are available on request to the corresponding author.

## Ethics Statement

The study was reviewed and approved by the Ethics Committee of the Psychology Department of Capital Normal University. Written informed consent was obtained from all adult participants and from the parents/legal guardians of all non-adult participants.

## Author Contributions

YW and ZS designed the experiments. JZ collected and analyzed the data. QM wrote the manuscript. XS and QM revised the manuscript.

## Conflict of Interest

The authors declare that the research was conducted in the absence of any commercial or financial relationships that could be construed as a potential conflict of interest.
