# Supplementary material for: Influence of Mothers/Grandmothers Coviewing Cartoons With Children on Children’s Viewing Experience
Source: Front Psychol. 2020 Jun 18;11:1232. doi: 10.3389/fpsyg.2020.01232 (PMC7326029; doi:10.3389/fpsyg.2020.01232)
Supplement: Supplementary file 1 [file Data_Sheet_1.PDF]

Appendix 1

Parents report

Dear parents:

We want to know your attitude towards children watching cartoons through the following questions. After the investigation, we will give you feedback on the characteristics of children's physical and mental development and the results of the survey. Hoping that we can promote the positive and healthy leading role of cartoons in children's growth. **Please answer the following questions in accordance with the actual situation, your personal information will be strictly confidential.** Thank you again for your support!

Children Information:

Name:_______ **Date of Birth:_______** **Gender:** **A** Male **B** Female

1. Your age:_______

2. Your occupation (please write specific, such as "university teacher", "taxi driver")

3. Your education level

A. Elementary school **B.** Junior high school **C.** High school or secondary school

D. University (specialist or undergraduate) **E.** Graduate student

4. Your annual income is ( )

A.30,000￥or less **B.**30,000-50,000￥ **C.**50,000-70,000￥

D.70,000-100,000￥ **E.**100,000-200,000￥ **F.** 200,000￥or more

5. Do you think your children are watching cartoons and have a big influence on them?

1————————2————————3————————4

No influence little influence more influence very big influence

6. Do you have restrictions on the time your child can watch cartoons?

1————————2————————3————————4

Strict limit often limit little limit no limit

7. Do you have restrictions on the content of your child watching cartoons?

1————————2————————3————————4

Strict limit often limit little limit no limit

Appendix 1

家长调查

尊敬的家长：

我们希望知道您对待儿童观看动画片的态度问题通过以下几道题。调查结束后我们会结合儿童身心发展特点和调查结果给您反馈，以促进动画片在儿童成长中发挥积极健康的引领作用。**请您按照真实情况回答下面问题，资料不会对外公布，请您放心填写。**再次对您的参与表示由衷的感谢！

儿童信息：姓名：_______ 出生日期：______年_____月 性别：A 男 B 女

1. 您的年龄_______

2. 您的职业（请写具体，比如“大学老师”、“出租车司机”）_______________

3. 您的文化程度________

   A. 小学 B. 初中 C. 高中或中专 D. 大学（专科或本科） E. 研究生

4. 您的年收入是（ ）

   A.3 万元以下 B.3-5 万元 C.5-7 万元

   D.7-10 万元 E.10-20 万元 F.20 万元以上

5. 您觉得孩子看动画片，对他们的身心健康有影响吗？

   1————————2————————3————————4

   没有影响 影响很小 影响较大 影响很大

6. 您是否会对孩子看动画片的时间有所限制？

   1————————2————————3————————4

   严格限制 经常限制 很少限制 没有限制

7. 您是否会对孩子看动画片的内容有所限制？

   1————————2————————3————————4

   严格限制 经常限制 很少限制 没有限制
