# Supplementary material for: Influence of Mothers/Grandmothers Coviewing Cartoons With Children on Children’s Viewing Experience
Source: Front Psychol. 2020 Jun 18;11:1232. doi: 10.3389/fpsyg.2020.01232 (PMC7326029; doi:10.3389/fpsyg.2020.01232)
Supplement: Supplementary file 2 [file Data_Sheet_2.PDF]

Children's cartoon viewing experience questionnaire

Name： __________ **gender：** _______ **age：** _______

Dimension	Question	Coding scheme	Coding explanation
**Understand**	1. Why is Briar's ass hurt?	**2 points:** Because he wants to eat honey, the fire is to use smoked bees, the butt is burned.<br>**1 point:** Did not answer the reason for the incident. Only answered "the ass was burned."<br>**0 point:** “I don't know” or the answer doesn't match the question.	This dimension mainly examines the child's understanding of the cartoon plot and content.<br>The higher the child's score, the better their understanding.
	2. Why is Bramble climbing the ladder?	**2 points:** Because he wants to eat honey, but the honeycomb is too high, he can't reach it.<br>**1 point:** There is no answer to the cause of the incident, just answer "He can't reach" or "Briar let him climb".<br>**0 point:** “I don't know” or the answer doesn't match the question.	
	3. Where is Logger Vick's rocket？	**2 points:** It fell out and was taken away by Warren.<br>1 point: Lost.<br>**0 point:** “I don't know” or the answer doesn't match the question.	
	4. Why did Logger Vick still not beat Briar and Bramble after finding the rocket?	**2 points:** Logger Vick missed the shot because Warren was holding the rocket.<br>**1 point:** Only answer some minor reasons like "Bears ran away, shot in the sky", etc.<br>**0 point:** “I don't know” or the answer doesn't match the question.	
**Identity**	5.Who do you like the most in cartoons? Why?	**A.** Logger Vick (1 point)<br>**B.** Briar (2 points)<br>**C.** Bramble (3 points)<br>**D.** Warren-squirrel (4 points)<br>**E.** Others (5 points) reason__________________	This dimension mainly examines which animated character children are most fond of, and whether they will beautify the character's violence because of this identity.
	6. Who do you hate most about cartoons? why?	**A.** Logger Vick (1 point)<br>**B.** Briar (2 points)<br>**C.** Bramble (3 points)<br>**D.** Warren (4 points)<br>**E.** Others (5 points) reason_____________	
	7. Logger Vick is always hit by Briar and Bramble. Do you think he is pitiful? why?	**A.** No pity, this is the punishment he deserves (1 point)<br>**B.** Poor, but also the punishment he deserves (2 points)<br>**C.** Poor (3 points)<br>**D.** “I don't know” or the answer doesn't match the question. (4 points)	

**Reality**	8. To eat honey, Briar and Bramble went to climb the tree and set the fire. Do you think this could happen in real life? Why?<br>9. When Bramble was chased by a bee, he ran away on a ladder that had been broken into two. Do you think this will happen in real life? why?<br>10. Logger Vick finally used the rocket to shoot Warren into the sky. Do you think this kind of thing will happen in real life? why?	**Do you think this could happen in real life?**<br>**A.** Yes (1 point)<br>**B.** No (2 points)<br>**C.** “I don’t know” or “I am not sure” (3 points)<br><br>**Why?**<br>**A. Experience proof** (1 point)<br>**B. Hypothesis proof** (2 points)<br>**C. Redundant proof** (3 points)<br>**D. Don’t know.** (4 points)	This dimension mainly examines whether children can distinguish between reality and illusory events, and whether they think that cartoon scenes can happen in real life, and what interpretation strategies they will use.
**Emotion**	11. What do you think of when Logger Vick fights with Briar and Bramble? Are you happy or scared?<br>12. Do you think your friends will feel nervous and scared when they watch this cartoon?<br>13. What is your most impressive sentence?<br>14. What is your most impressive scene？	**A.** Happy (1 point)<br>**B.** Fear (2 points)<br>**C.** Happy and scared (3 points)<br>**D.** Not happy or afraid (4 points)<br>**E.** Other feelings (5 points)<br>**A.** No (1 point)<br>**B.** They will feel a little fear (2 points)<br>**C.** Yes (3 points)<br>**D.** “I don't know” (4 points).<br><br>The emotional state of the character who said this sentence<br>**A.** Positive (1 point)<br>**B.** Neutral (2 points)<br>**C.** Negative (3 points)<br>**D.** “Not remembered” (4 points)<br>**A.** Danger (1 point)<br>**B.** Violence (2 points)<br>**C.** Other (3 points)<br>**D.** Don’t know or Not remembered (4 points)	This dimension mainly examines whether children will feel uneasy or excited when watching cartoons in certain violent scenes. Whether children are more impressed with violence and aggressive scenes and language.<br><br>The higher the child's score, the more positive emotions (such as excitement) that children are awakened by violent scenes. The lower the child's score, the more the reverse emotions (such as restlessness) are awakened.

**Aggressive Fantasy**	15. If you are a Briar, how would you teach Logger Vick a lesson?	**A.** Physical attack (1 point)<br>**B.** Speech attacks (2 points)<br>**C.** Alternative attacks (3 points)<br>**D.** No attack or can be ignored (4 points)	This dimension is primarily a measure of a child's aggressiveness because it is also associated with realistic aggression. The higher the child's score, the more aggressive illusions they have.
	16. If you can be an animal in a cartoon, what kind of animal do you want to be? why?	**2 points:** carnivores and aggressive animals such as tigers<br>**1 point:** non-carnivorous animals<br>**0 point**：“I don't know”.	

儿童动画片收视体验问卷

姓名：__________　　　　　　　　性别：_______　　　　　　年龄：_______

维度	问题	编码计分	分数解释
理解	1 熊大的屁股为什么会受伤？	2 分：想吃蜂蜜，烧火用烟熏蜜蜂，被火烧的<br>1 分：没有回答引发原因，只回答被火烧的<br>0 分：不知道或答非所问	了解孩子对动画片情节、内容的理解程度。得分越高，理解程度越高。
	2 熊二为什么要爬梯子？	2 分：想吃蜂蜜，但是蜂窝太高了，够不着<br>1 分：够不着（没有回答引发原因），熊大让他爬<br>0 分：不知道或答非所问	
	3 光头强的火箭弹呢？	2 分：掉出来了，被萝卜头捡走了<br>1 分：掉了<br>0 分：不知道或答非所问	
	4 为什么光头强找到了火箭弹还是没有打败熊大熊二？	2 分：萝卜头把火箭弹当成它的萝卜，抱着不放手，光头强射偏了<br>1 分：小熊熊逃跑了，射到天上去了等次要原因<br>0 分：不知道或答非所问	
认同	5 你最喜欢动画片里的谁呀？为什么？	A.光头强（1 分）<br>B.熊大（2 分）<br>C.熊二（3 分）<br>D.萝卜头（4 分）<br>E.其他（5 分）　理由____________	主要考察儿童对哪个动画角色最为喜爱，是否会因为这种认同而美化角色的暴力行为。
	6 你最讨厌动画片里的谁呢？为什么？	A.光头强（1 分）<br>B.熊大（2 分）<br>C.熊二（3 分）<br>D.萝卜头（4 分）<br>E.其他（5 分）　理由____________	
	7 光头强总是被熊大熊二打，你觉得它可怜吗？为什么？	A.不可怜，应得的（1 分）<br>B.可怜，但是应得的（2 分）<br>C.可怜（3 分）<br>D.不知道或答非所问（4 分）	
现实感	8 熊大熊二为了吃蜂蜜，又是爬树又是烧火，你觉得这在生活中会不会真的发生？为什么？	**你认为这件事会不会发生？**<br>A.会（1 分）<br>B.不会（2 分）	主要考察儿童是否能区分现实与虚幻，是否会认为动画片的场景能在现实生活中发生，

	9 熊二被蜜蜂追时，踩着断成两截的梯子逃跑，你觉得这在生活中会不会真的发生？为什么？<br><br>10 光头强最后用火箭弹把萝卜头射到天上去了，你觉得这样的事在生活中会不会真的发生吗？为什么？	**为什么？**<br>A.经验证明（1 分）<br>B.假设证明（2 分）<br>C.多余证明（3 分）<br>D.不知道（4 分）	以及他们都会使用什么解释策略。
**情绪**	11 你看到光头强和熊大熊二打架时是什么心情呢？是高兴还是害怕？	A.高兴（1 分）<br>B.害怕（2 分）<br>C.又高兴又害怕（3 分）<br>D.不高兴也不害怕（4 分）<br>E. 其他（5 分）	主要了解儿童在观看一定暴力场景动画片时是会感到不安还是兴奋？是否会对暴力和攻击性的场面、语言更加印象深刻。<br>分数越高，表示暴力场景的正向情绪（比如兴奋）唤醒更高，分数低表示反向情绪（比如不安）唤醒高。
	12 你觉得你的朋友在看这个动画片时会不会感到紧张和害怕？	A.不会（1 分）<br>B.会有一些害怕（2 分）<br>C.会（3 分）<br>D.不知道（4 分）	
	13 你印象最深的一句话是什么？	说这句话的角色的情绪状态<br>A.积极（1 分）<br>B.中性（2 分）<br>C.消极（3 分）<br>D.不记得（4 分）	
	14 你印象最深的一个画面是什么？	A.危险（1 分）<br>B.暴力（2 分）<br>C.其他（3 分）<br>D.不知道/不记得（4 分）	
**攻击性幻想**	15 如果你是熊大，你会怎么教训光头强呢？	A.身体攻击（1 分）<br>B.言语攻击（2 分）<br>C.替代性攻击（3 分）<br>D.没有攻击或可以忽略（4 分）	攻击性幻想是测量攻击性的指标，它与现实的攻击性行为也相关。得分越高，攻击性幻想越高。
	16 如果你可以成为动画片里的一种动物，你想成为哪种动物呢？为什么？	2 分：肉食动物以及攻击性较强的动物，比如老虎<br>1 分：非肉食动物<br>0 分：不知道	
